# *PDCD1* Polymorphisms May Predict Response to Anti-PD-1 Blockade in Patients With Metastatic Melanoma

**DOI:** 10.3389/fimmu.2021.672521

**Published:** 2021-06-09

**Authors:** Sagun Parakh, Ashan Musafer, Sabrina Paessler, Tom Witkowski, Connie S. N. Li Wai Suen, Candani S. A. Tutuka, Matteo S. Carlino, Alexander M. Menzies, Richard A. Scolyer, Jonathan Cebon, Alexander Dobrovic, Georgina V. Long, Oliver Klein, Andreas Behren

**Affiliations:** ^1^ Medical Oncology Unit, Austin Health, Melbourne, VIC, Australia; ^2^ Olivia Newton-John Cancer Research Institute, Melbourne, VIC, Australia; ^3^ La Trobe University School of Cancer Medicine, Melbourne, VIC, Australia; ^4^ Department of Mathematics and Statistics, La Trobe University, Melbourne, VIC, Australia; ^5^ Department of Medical Oncology, Westmead and Blacktown Hospitals, Sydney, NSW, Australia; ^6^ Melanoma Institute Australia, The University of Sydney, North Sydney, NSW, Australia; ^7^ Department of Medical Oncology, Royal North Shore and Mater Hospitals, Sydney, NSW, Australia; ^8^ Tissue Pathology and Diagnostic Oncology, Royal Prince Alfred Hospital and NSW Health Pathology, Sydney, NSW, Australia; ^9^ Faculty of Medicine and Health, The University of Sydney, Sydney, NSW, Australia; ^10^ Department of Clinical Medicine, Macquarie University, Sydney, NSW, Australia; ^11^ Department of Medicine, University of Melbourne, Melbourne, VIC, Australia

**Keywords:** metastatic melanoma, PD1, polymorphism, predictive biomarker, immunotherapy

## Abstract

A significant number of patients (pts) with metastatic melanoma do not respond to anti-programmed cell death 1 (PD1) therapies. Identifying predictive biomarkers therefore remains an urgent need. We retrospectively analyzed plasma DNA of pts with advanced melanoma treated with PD-1 antibodies, nivolumab or pembrolizumab, for five PD-1 genotype single nucleotide polymorphisms (SNPs): PD1.1 (rs36084323, G>A), PD1.3 (rs11568821, G>A), PD1.5 (rs2227981, C>T) PD1.6 (rs10204225, G>A) and PD1.9 (rs2227982, C>T). Clinico-pathological and treatment parameters were collected, and presence of SNPs correlated with response, progression free survival (PFS) and overall survival (OS). 115 patients were identified with a median follow up of 18.7 months (range 0.26 – 52.0 months). All were Caucasian; 27% BRAF V600 mutation positive. At PD-1 antibody commencement, 36% were treatment-naïve and 52% had prior ipilimumab. The overall response rate was 43%, 19% achieving a complete response. Overall median PFS was 11.0 months (95% CI 5.4 - 17.3) and median OS was 31.1 months (95% CI 23.2 - NA). Patients with the G/G genotype had more complete responses than with A/G genotype (16.5% *vs.* 2.6% respectively) and the G allele of PD1.3 rs11568821 was significantly associated with a longer median PFS than the AG allele, 14.1 *vs.* 7.0 months compared to the A allele (p=0.04; 95% CI 0.14 – 0.94). No significant association between the remaining SNPs and responses, PFS or OS were observed. Despite limitations in sample size, this is the first study to demonstrate an association of a germline PD-1 polymorphism and PFS in response to anti-PD-1 therapy in pts with metastatic melanoma. Extrinsic factors like host germline polymorphisms should be considered with tumor intrinsic factors as predictive biomarkers for immune checkpoint regulators.

## Introduction

Programmed cell death-1 (PD-1) is a member of the CD28 family of co-stimulatory molecules and is expressed on activated CD4^+^ and CD8^+^ T cells (1). Upon binding to its ligand, programmed death-ligand 1 and 2 (PD-L1 and PD-L2), PD-1 is responsible for negatively regulating the effector phase of T-cell responses and maintaining immune tolerance (2). Constitutive high level expression of PD-1 on tumor specific T lymphocytes is a major factor restraining an effective anti-tumor immune response in patients with advanced malignancies (3). The monoclonal antibodies targeting PD-1, nivolumab and pembrolizumab, have shown impressive and durable responses in cancer patients resulting in regulatory approval in many cancer subtypes. Despite the success of these agents, a significant proportion of patients do not respond to anti-PD-1 therapy; therefore identifying biomarkers that predict therapeutic efficacy remains an urgent need (4). The human gene for PD-1, *PDCD1*, is localized on chromosome 2q37 (5). A number of single nucleotide polymorphisms (SNPs) in *PDCD1* have been identified and shown to be associated with the development of autoimmune conditions, including Crohn’s disease, systemic lupus erythematosus, type I diabetes, rheumatoid arthritis, and multiple sclerosis (6). In addition, certain PD-1 polymorphisms have been shown to be associated with an improved viral control in patients with chronic viral infections (7). The effect of PD-1 polymorphism in cancer remains unclear, with some studies reporting an increase in the risk of developing some cancer types while others have reported a reduced risk (8–10). Despite the crucial role that the PD-1/PD-L1 pathway plays in limiting anti-tumor immune responses, there are no data exploring the potential influence of PD-1 polymorphisms on the treatment response to anti-PD-1 blockade.

In this study we retrospectively evaluated the association of polymorphisms in *PDCD1* with responses and survival in patients with metastatic melanoma treated with anti-PD-1 monoclonal antibodies. We demonstrate that certain PD-1 SNPs may be associated with improved anti-melanoma outcomes after immunotherapy and can potentially serve as biomarkers.

## Methods

### Patients

Patients with metastatic melanoma treated with single agent anti-PD-1 antibodies, nivolumab or pembrolizumab, between January 2014 and June 2016 in three major Australian melanoma centers were studied. Data collected included: baseline demographics, disease stage, disease characteristics; details of PD-1 inhibitor treatment (type, dosage, number of cycles received); prior systemic treatments; and time to endpoint data. End-points evaluated were overall response rate (ORR; defined as CR or PR), progression free survival (PFS) and overall survival (OS). PFS was defined as time between date of commencement of therapy to date of progression or death. Response assessments were made as per the Response Evaluation Criteria in Solid Tumors (RECIST) v1.1 (11).

### Genotyping

The SNPs selected for study were those either located in the promoter or untranslated region or coding region of the gene, those previously evaluated in relation to cancer, or those with evidence of functional significance in autoimmune diseases. DNA extracted from baseline blood samples were analyzed by polymerase chain reaction (PCR) and high-resolution melt (HRM) analysis on the Rotor-Gene 6000 (Corbett Life Science). A 20µL reaction mixture contained; 1X PCR Buffer, 2.5mM MgCl_2_, 800nM total of dNTPs, 250nM forward primer, 250nM reverse primer, 5 µM of SYTO9 intercalating dye (Invitrogen), 0.5 U of HotStarTaq polymerase (Qiagen), 2 ng of genomic DNA and UltraPure™ PCR grade water (ThermoFisher Scientific). The cycling and melting conditions for all PD-1 SNP genotyping assays were as follows; 15 min activation at 95°C followed by 55 PCR cycles of 98°C for 20 seconds, 65°C for 30 seconds and 72°C for 25 seconds, then a post PCR hold at 98°C for 1 min followed by high resolution melting from 68°C to 98°C, 0.2°C per step. All samples were tested in duplicate and analyzed using the Rotor-gene 6000 software.

The study was approved by individual institution ethics committees of Austin Health, Melbourne, Westmead Hospital, Royal North Shore and Mater Hospitals, Sydney and patients either prospectively consented to inclusion or consent was waived as per individual institution ethics committee guidance.

**Table:** 

SNP	rs Number	Primer	Primer Sequence
PD1.1	rs36084323	PD1.1 F	5’-TTAGCCATGGACAGTTGTCATTCAG-3’
		PD1.1 R	5’- GTGCCTGGCCTCTGCCTTC-3’
PD1.3	rs11568821	PD1.3 F	5’-GGCAGCAACCTCAATCCCTA-3’
		PD1.3 R	5’- AGGCAGGCACACACATGG-3’
PD1.5	rs2227981	PD1.5 F	5’-GCGGAATGGGCACCTCATC-3’
		PD1.5 R	5’- CAAGAGCAGTGTCCATCCTCA -3’
PD1.6	rs10204225	PD1.6 F2	5’-GTGTTGGGAGGGCAGAAGT-3’
		PD1.6 R2	5’- TTTCAGGAATGGGTTCCAAG -3’
PD1.9	rs2227982	PD1.9 F	5’-GCTGACTCCCTCTCCCTTTC-3’
		PD1.9 R	5’- ATCCAGCTCCCCATAGTCCA -3’

### Statistical Analysis

Kaplan-Meier estimates of PFS and OS from commencement of immune checkpoint therapy were calculated separately for each individual SNP. Hazard ratios and corresponding 95% confidence intervals were estimated through univariate and multivariate Cox proportional hazard models. Multivariate logistic regression analysis was used to explore the association between responses and SNP genotype. We performed Fisher’s exact tests per SNP genotypes to assess if responses observed between different SNP genotypes was significant. All analyses were carried out using R version 3.4.0 (12).

## Results

### Demographics and Efficacy Analyses

A total of 115 patients received either pembrolizumab or nivolumab between January 2014 and June 2016. The median follow-up after commencement of anti-PD-1 therapy was 18.7 months (range 0.26 – 52.0 months). The median age was 71.3 years (29.4 – 92.4), all patients were Caucasian and BRAF V600 mutations were detected in 24% of patients, while NRAS mutations were detected in 25% (**Table 1**). Thirty-six percent of patients were treatment naïve, 52% being treated with prior ipilimumab. The genotype frequency of SNPs evaluated in this study is consistent those reported in Caucasian populations (13) (**Table 2**).

**Table 1:** Demographics.

Demographics	N (%)
Number (N)	115 (100)
Median age - years (range)	71.3 (29.4 – 92.4)
Race	
Caucasian	115 (100)
AJCC stage	
IIIc	7 (6)
M1a	6 (5)
M1b	10 (9)
M1c	89 (77)
Not recorded	3 (3)
Mutational status	
BRAF V600 mutated	28 (24)
NRAS mutations*	24 (25)
Prior systemic treatment for metastatic disease	
No prior treatment	41 (36)
Ipilimumab	59 (51)
Dabrafenib and Trametinib	18 (16)
Chemotherapy	13 (11)

*Documented in 95 patient.

**Table 2:** PD-1 SNPs.

SNP	Polymorphism	Location	Ancestral allele	MAF	Genotype	Genotype frequency (%)	
						Control^*^	Study
PD1.1	rs36084323	promoter	G	0.15 (T)	G/GA/GA/A	97 3 0	99 1 0
PD1.3	rs11568821	intron	C	0.04 (T)	A/AA/GG/G	2 20 78	0 28 72
PD1.5	rs2227981	synonymous	G	0.35 (A)	C/CC/TT/T	38 44 18	46 48 6
PD1.6	rs10204225	3’UTR	G	0.03 (A)	A/A A/G G/G	0 0 100	0 20 80
PD1.9	rs2227982	missense	G	0.14 (A)	T/T C/T C/C	0 3 97	0 5 95

MAF, Minor allele frequency; SNP, Single-nucleotide polymorphism; UTR, untranslated regions.

*Caucasian population.

In our study the ORR was 43% with 22 patients (19%) obtaining a complete response. The median PFS was 11.0 months (95% CI 5.4 - 17.3) and median OS was 31.1 months (95% CI 23.2 - NA).

### Association Between Genotypes and Clinical Outcome

**Figure 1:**
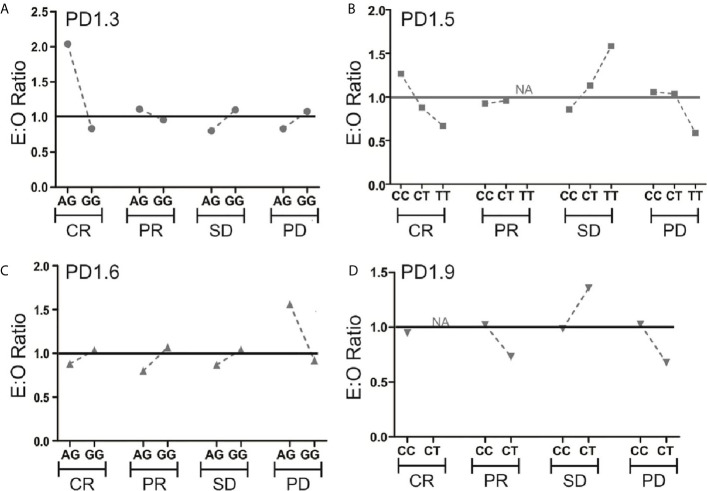
Expected (E)-to-observed (O) ratio of responses according to PD-1 SNP. Responses for all 4 SNPs **(A–D)** are shown as complete response (CR), partial response (PR), stable disease (SD) or progressive disease (PD) by dividing number of expected responses by number of observed responses within each genotype. The solid line at y=1 represents a theoretical E:O ratio of 1:1. NA signifies data points where one genotype had 0 events.

**Figure 2:**
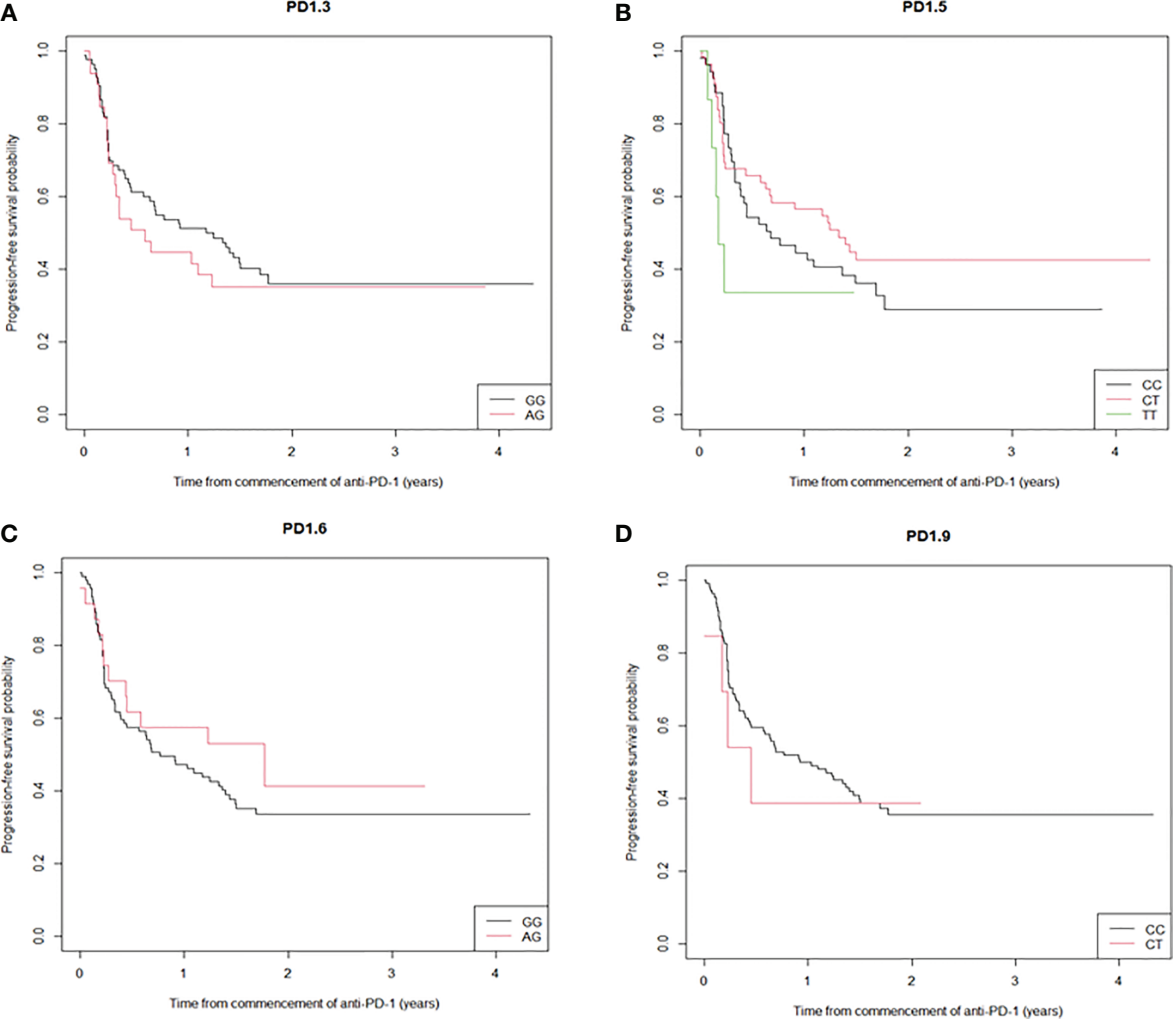
Kaplan-Meier curves of progression-free survival stratified by the individual SNPs. **(A)** the median PFS for PD1.3 rs11568821 GG allele was 14.1 months versus 7.0 months for the AG allele (HR 0.836 (95%CI (0.50-1.39) p=0.49); **(B)** the median PFS for PD1.5 rs2227981 CT allele 16 months (HR 0.79 (95%CI (0.48-1.27) p=0.329), 2.1 months for the TT allele (HR 1.78 (95%CI (0.70-4.57) p=0.228) versus 8.1 months for the CC allele; **(C)** the median PFS for PD1.6 rs10204525 21.3 months for the AG allele versus 9.2 months for the GG allele (HR 0.75 (95%CI (0.40-1.40) p=0.364); **(D)** the median PFS for PD1.9 rs2227982 CC allele was 11.0 months versus 5.4 months for the CT allele (HR 0.73 (95%CI (0.27-2.01) p=0.543).

Five PD-1 SNPs were genotyped in this cohort of patients with metastatic melanoma treated with an anti-PD-1 inhibitor. Almost a third of patients, 32 (28%), had the PD1.3 rs11568821 A/G genotype and 83 (72%) the G/G genotype. Patients with the G/G genotype had more complete responses than with A/G genotype (16.5% *vs.* 2.6%) respectively (**Figure 1**). No significant association between the remaining SNPs and responses were observed or between SNP genotypes and response (**Supplementary Table 1**). Kaplan-Meier estimates of PFS were calculated for each SNP (**Figure 2**) as well as separately for each PD1.3 genotype (**Supplementary Figure 1**). On univariate analysis, none of the PD-1 SNPs evaluated influenced PFS or OS, however in a multivariate Cox regression model (**Supplementary Table 2**) including AJCC stage and an interaction term with age, the presence of the G/G PD1.3 SNP was significantly associated with an improved PFS (p= 0.04).

## Discussion

This is the first study to evaluate the association between response to PD-1 inhibitors and germline polymorphisms in *PDCD1* in patients with metastatic melanoma. The ORR in our patient cohort was similar to those reported in clinical trials of anti-PD1 therapies in metastatic melanoma (14, 15). We identified that the G allele of PD1.3 rs11568821 was significantly associated with longer PFS in anti-PD-1 treated patients. Germline SNPs in immune-regulatory genes have been widely conducted, but mainly in the context of cancer-risk, as nicely summarized by Wagner et al. (16). Only a handful of studies have linked responses to immunotherapy or the advent of immune-related adverse events (irAEs) during treatment to SNPs in various immune-regulatory genomic regions, including *CTLA4* and *PDCD1* (17–20). The *PDCD1* SNPs PD1.5 (rs2227981) and PD1.9 (rs2227982) are both located within exon 5 of the *PDCD1*. PD1.5 is a synonymous polymorphism while PD1.9 is a non-synonymous SNP resulting in the amino acid substitution from valine to alanine at codon 215. The PD1.1 SNP G/A (rs36084323) is located in the promoter region, and was very uncommon in our patient population as previously reported (21). PD1.3 (rs11568821) is a guanine (G) to adenine (A) SNP in intron 4 at nucleotide +7146. This substitution alters the binding of runt-related transcription factor 1 (RUNX1) transcription factors affecting transcriptional regulation and PD-1 expression (21–24). Additionally this SNP results in impaired PD-1-mediated inhibition of T cell proliferation and interferon gamma (IFNγ) production and augments lymphocyte activity (25). Our findings suggest that aberrant regulation of PD-1 expression leads to the observed increased efficacy of anti-PD-1 therapy in patients carrying this distinct polymorphism. In keeping with such a mechanism pre-clinical work demonstrates that a lower PD-1 expression level on tumor specific T lymphocytes is predictive for a response to anti-PD-1 blockade in mouse tumor models (26). It could also be speculated that the frequency of exhausted PD-1^+^ T cell subpopulations that can be reinvigorated by anti-PD-1 blockade may differ between individuals based on the presence of certain PD-1 germline polymorphisms but functional effects of PD1.3 in the cancer setting remains to be established. Of interest, several population-based studies conducted largely in the Asian population and meta-analysis of available data have shown a significantly lower cancer risk associated with the PD1.3 (rs11568821) SNP (8, 27, 28), while other studies could not confirm these findings (29). The risk for the development of melanoma and the here examined SNPs is unclear, and we have not been able to find well annotated WGS data in sufficiently large cohorts of melanoma patients to cross-reference observed-to-expected minor allele frequency (MAF) as derived from the 1000 Genomes project. TCGA is providing healthy tissue WGS data from only minor sample populations, WGS data from healthy tissue, and even the recently published Pan-cancer analysis of whole genomes provides only information for a small set of melanoma patients, not all of which are of Caucasian origin (30). Multiple other limitations for GWAS studies exist, including lifestyle and additional factors that may contribute to cancer risk and most of these limitations are true for our study as well. The MAF frequencies as listed in **Table 2** demonstrate that a very large patient number beyond the here presented one would be necessary for a sufficiently powered (0.8) study. Additionally, the number of SNPs within each study needs to be corrected for further increasing the necessary sample size for significance. Hence, the here detected associations (and non-associations), while interesting, need confirmation in much larger cohorts, and efforts to establish these are currently underway (31). Another additional challenge is to distinguish between predictive vs prognostic associations, hence study control arms (where available from historical trials) would need to be typed for SNP occurrence as well.

So far efforts to identify predictive biomarkers to anti-PD-1 therapy have mainly focused on tumor intrinsic factors. PD-L1 expression on melanoma cells has shown to enrich for responders to treatment with anti-PD-1 antibodies however in all studies evaluating anti-PD-1 therapies, a significant proportion of PD-L1 negative patients benefitted from treatment (32–34). This can be explained by the spatial and temporal heterogeneous PD-L1 expression in tumor tissue and the discordance in PD-L1 expression between metastatic tissue of the same patient (35, 36). Furthermore, apart from inherent technical issues with IHC, varying IHC cut-offs to define PD-L1 positivity, oncogenic versus induced PD-L1 expression, staining of tumor versus immune cells and the dynamic nature of PD-L1 expression complicates its use as a reliable biomarker (37, 38). Similarly patients with tumors that harbor higher numbers of non-synonymous single nucleotide polymorphisms leading to an increased number of neoepitopes have a trend to a better response to anti-PD-1 treatment (39). In addition, clinical pathological factors including peripheral blood markers (38, 40) were also be shown of potential predictive value. More recently it has been shown that the composition of intestinal microbiome can influence the response to anti-PD-1 therapy (41). Given the complexity of anti-tumor immune responses it is now recognized that no single biomarker can predict the success to anti-PD-1 blockade. Nonetheless a variety of factors that are associated with more or less favorable outcomes are being identified and aggregating such tumor-intrinsic features and tumor-extrinsic factors is likely to lead to models (or algorithms) that can assist identifying those patients most likely to respond to anti-PD-1 therapy and those likely to need alternative approaches including combination immunotherapies. Our study identifies the G allele of PD1.3 rs11568821 as the potential first germline SNP biomarker for the efficacy of anti-PD-1 therapies.

In conclusion, this is the first study that potentially demonstrates an association of a germline PD-1 polymorphism and longer PFS in response to anti-PD-1 therapy in patients with metastatic melanoma. Unfortunately, no PBMCs are available from the study cohort, so we could not pinpoint therapy-predictive value of SNPs with PD-1 expression. Additionally, the relative short follow-up time and limited study cohort size (n=115) available for this study emphasizes the importance of confirmative study cohorts. Future studies should additionally include SNPs or SNV in other relevant genes that may influence response to anti-PD-1 treatment or combination immunotherapies that may be used upfront or sequentially if the initial therapy fails. Despite these limitations these results suggest that PD-1 rs11568821 may be a biomarker for identifying patients likely to benefit from anti-PD-1 therapies. Together with other immune-markers this will help in establishing patient-specific cancer- immunomaps for patient stratification and prediction of response.

## Data Availability Statement

The raw data supporting the conclusions of this article will be made available by the authors, without undue reservation.

## Ethics Statement

The study was approved by individual institution ethics committees of Austin Health, Melbourne, Westmead Hospital, Royal North Shore and Mater Hospitals, Sydney and patients either prospectively consented to inclusion or consent was waived as per individual institution ethics committee guidance. The ethics committee waived the requirement of written informed consent for participation.

## Author Contributions

AB, OK, AD, and SbP contributed to conception and design of the study. AB, OK and SgP wrote the manuscript. AD, CS, and AMu wrote sections of the manuscript. All authors contributed to sample collection, data gathering and analysis and manuscript revision. All authors contributed to the article and approved the submitted version.

## Conflict of Interest

MC has a consultant advisory role with BMS, MSD, Amgen, Novartis, Pierre Fabre, Roche, Sanofi, Merck and Co, Ideaya, Regeneron, Nektar, Eisai and Q biotics and OncoSec. AMM is a consultant advisor to BMS, MSD, Novartis, Roche, Pierre-Fabre, QBiotics. RAS has received fees for professional services from Qbiotics, Novartis, Merck Sharp & Dohme, NeraCare, AMGEN Inc., Bristol-Myers Squibb, Myriad Genetics, and GlaxoSmithKline. JC has sat on advisory boards for Novartis and GSK. GL is consultant advisor for Aduro Biotech Inc, Amgen Inc, Array Biopharma inc, Boehringer Ingelheim International GmbH, Bristol-Myers Squibb, Hexel AG, Highlight Therapeutics S.L., Merck Sharpe & Dohme, Novartis Pharma AG, OncoSec, Pierre Fabre, QBiotics Group Limited, Regeneron Pharmaceuticals Inc, SkylineDX B.V., and Specialised Therapeutics Australia Pty Ltd.

The remaining authors declare that the research was conducted in the absence of any commercial or financial relationships that could be construed as a potential conflict of interest.

## Supplementary Material

Supplementary Table 1 Relationship between responses vs genotype.

Supplementary Table 2 Multivariate Cox model for PFS.

Supplementary Figure 1 Kaplan-Meier curves of progression-free survival stratified by the individual genotype for SNP 1.3, GG allele versus AG allele respectively **(A)** Complete response **(B)** Partial response **(C)** Stable disease **(D)** Progressive disease.
