# Supplementary material for: PDCD1 Polymorphisms May Predict Response to Anti-PD-1 Blockade in Patients With Metastatic Melanoma
Source: Front Immunol. 2021 Jun 9;12:672521. doi: 10.3389/fimmu.2021.672521 (PMC8220213; doi:10.3389/fimmu.2021.672521)
Supplement: Supplementary Table 1 — Relationship between responses vs genotype. [file Table_1.docx]

**Supplementary Table 1: Relationship between responses vs genotype.**

| **SNP** | **Genotype** | **Response** | | | **No response/PD** |  |
| --- | --- | --- | --- | --- | --- | --- |
|  |  | **CR** | **PR** | **SD** |  |  |
| 1.3 | GG | 19 | 21 | 17 | 26 | p = 0.317 |
|  | AG | 3 | 7 | 9 | 13 |  |
| 1.5 | TT | 2 | 0 | 1 | 4 | P = 0.597 |
|  | CC | 8 | 14 | 14 | 17 |  |
|  | CT | 12 | 14 | 11 | 18 |  |
| 1.6 | AG | 5 | 7 | 6 | 5 | p = 0.563 |
|  | GG | 17 | 21 | 20 | 34 |  |
| 1.9 | CC | 22 | 26 | 25 | 36 | p = 0.724 |
|  | CT | 0 | 2 | 1 | 3 |  |

CR: complete response; PD: progressive disease; PR: partial response; SD: stable disease

**Supplementary Table 2: Multivariate Cox model for PFS**

| Factors | Hazard ratio | 95% confidence interval | p-value |
| --- | --- | --- | --- |
| Age | 0.96 | (0.92, 0.99) | 0.021 |
| PD1.3  AG  GG | Reference  0.05 | (0.003, 0.87) | 0.040 |
| AJCC stage  IIIc  M1a  M1b  M1c | Reference  0.82  1.26  1.75 | (0.16, 4.12)  (0.30, 5.33)  (0.55, 5.63) | 0.812  0.751  0.346 |
| Age:PD1.3 | 1.04 | (0.99, 1.08) | 0.061 |
