# Supplementary figures and images for: PDCD1 Polymorphisms May Predict Response to Anti-PD-1 Blockade in Patients With Metastatic Melanoma
Source: Front Immunol. 2021 Jun 9;12:672521. doi: 10.3389/fimmu.2021.672521 (PMC8220213; doi:10.3389/fimmu.2021.672521)

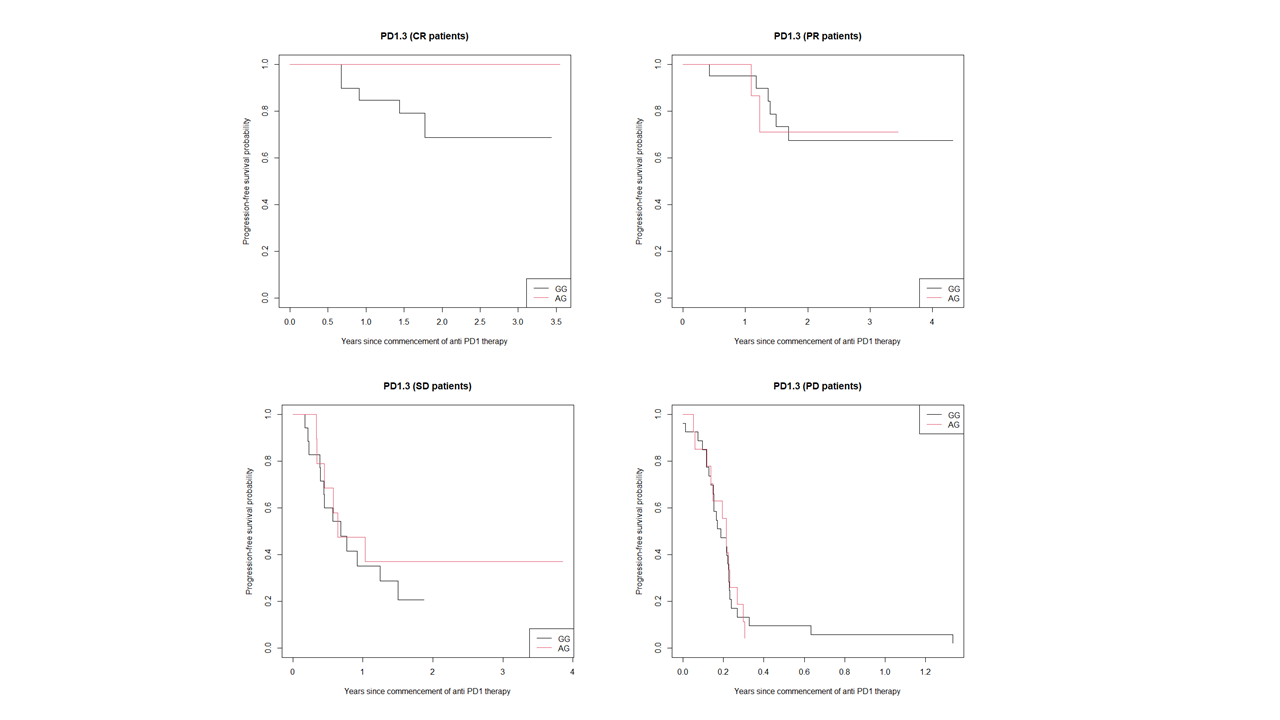

Supplement: Supplementary Figure 1 — Kaplan-Meier curves of progression-free survival stratified by the individual genotype for SNP 1.3, GG allele versus AG allele respectively (A) Complete response (B) Partial response (C) Stable disease (D) Progressive disease. [file Image_1.tif]
